# Dual-frequency piezoelectric micromachined ultrasound transducer based on polarization switching in ferroelectric thin films

**DOI:** 10.1038/s41378-023-00595-z

**Published:** 2023-10-02

**Authors:** Jin Soo Park, Soo Young Jung, Dong Hun Kim, Jung Ho Park, Ho Won Jang, Tae Geun Kim, Seung-Hyub Baek, Byung Chul Lee

**Affiliations:** 1 Bionics Research Center, Korea Institute of Science and Technology, Seoul, 02792 Republic of Korea; 2 Department of Electrical Engineering, Korea University, Seoul, 02841 Republic of Korea; 3 Center for Electronic Materials, Korea Institute of Science and Technology, Seoul, 02792 Republic of Korea; 4 Department of Material Science and Engineering, Seoul National University, Seoul, 08826 Republic of Korea; 5 Division of Bio-Medical Science and Technology, KIST School, University of Science and Technology (UST), Seoul, 02792 Republic of Korea; 6 KHU-KIST Department of Converging Science and Technology, Kyung Hee University, Seoul, 02447 Republic of Korea

**Keywords:** Electrical and electronic engineering, Physics, Materials science

## Abstract

Due to its additional frequency response, dual-frequency ultrasound has advantages over conventional ultrasound, which operates at a specific frequency band. Moreover, a tunable frequency from a single transducer enables sonographers to achieve ultrasound images with a large detection area and high resolution. This facilitates the availability of more advanced techniques that simultaneously require low- and high-frequency ultrasounds, such as harmonic imaging and image-guided therapy. In this study, we present a novel method for dual-frequency ultrasound generation from a ferroelectric piezoelectric micromachined ultrasound transducer (PMUT). Uniformly designed transducer arrays can be used for both deep low-resolution imaging and shallow high-resolution imaging. To switch the ultrasound frequency, the only requirement is to tune a DC bias to control the polarization state of the ferroelectric film. Flextensional vibration of the PMUT membrane strongly depends on the polarization state, producing low- and high-frequency ultrasounds from a single excitation frequency. This strategy for dual-frequency ultrasounds meets the requirement for either multielectrode configurations or heterodesigned elements, which are integrated into an array. Consequently, this technique significantly reduces the design complexity of transducer arrays and their associated driving circuits.

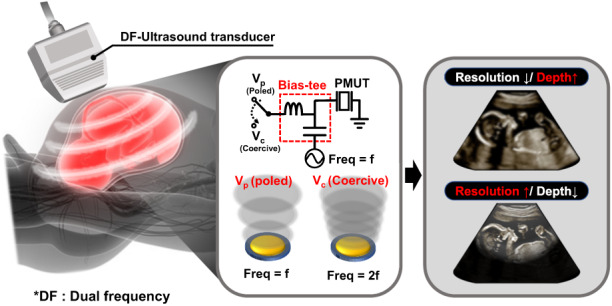

## Introduction

Multifrequency ultrasound devices provide many benefits and are used extensively in medical clinical applications, such as multiscale imaging, harmonic contrast agent imaging, and image-guided high-intensity focused ultrasound (HIFU)^1,2^. They are especially suitable for biomedical ultrasound imaging, as the sonographer can carefully select a frequency to tune the penetration depth and spatial resolution. This ability to achieve both good penetration depth and high resolution provides a comprehensive understanding of the full anatomic information of the target, helping the clinicians’ diagnosis^3^. Compared to conventional ultrasound, which operates within a predefined frequency band, dual-frequency ultrasound is capable of effectively enhancing the contrast of the produced image. Moreover, nonlinear oscillations of microbubbles, which are used as a contrast agent for angiography under exposure to dual-frequency ultrasound, reduce the threshold value^4^ for acoustic cavitation and generate additional frequency responses, such as harmonics, sub-harmonics, and ultra-harmonics^5,6^. By extracting harmonic signals from the backscattered echo, the isolation of the nonlinear response of the microbubbles from that of human soft tissue is possible and results in improved images for vascular remodeling^7^.

Traditional single-frequency operating devices cannot meet the requirements of these applications. Accordingly, multifrequency (especially dual-frequency) transducers have been proposed as a promising solution. The conventional approach to achieving dual-frequency ultrasound devices is to integrate the high- and low-frequency operating elements in either a vertical or horizontal configuration^8^. In a bilayered stack, each element is fabricated with a different thickness to determine the frequency band, and then they are sequentially bonded with one underneath the other. However, if both layers in this vertically stacked structure are fabricated from high-property piezoelectric materials (such as Pb(Zr, Ti)O_3_ (PZT) and Pb(Mg_1/3_Nb_2/3_)O_3_–PbTiO_3_ (PMN-PT)), there are significant coupling issues between the two layers that can generate aliasing echoes, which shift the resonant frequencies of both layers. To prevent this phenomenon, a frequency-selective anti-matching layer needs to be placed between the top and bottom layers to provide isolation^8–10^. Moreover, acoustic matching for both the low and high frequencies is difficult to optimize^11^, and fabrication becomes more difficult as the transducer dimensions are scaled down. One alternative solution is the interleaved array; this is referred to as a horizontal stack, where the low-frequency elements are laterally positioned on both sides of a central high-frequency element^12^. Here, even-numbered elements are used for the transmission of the ultrasound, and odd-numbered elements are used for reception. This technique does not require an anti-matching layer and does not have to modify the initial performance of the subarray. However, these horizontally arranged elements cause overlapping of the transmission and receiving beams and increase the footprint compared to regular array designs^1^.

The inherent manufacturing challenges with stacked arrays have inspired developers to investigate microelectromechanical system (MEMS)-based devices, such as capacitive micromachined ultrasound transducers (CMUTs) and piezoelectric micromachined ultrasound transducers (PMUTs). These microfabrication techniques enable the monolithic integration of each designed subarray with different frequency bands^13–15^ and unique flexural vibration of the membranes, initiating studies on the achievement of multifrequency ultrasound operation based on uniform element transducer arrays^16–18^. In MUTs, a well-known approach for achieving multifrequency ultrasound is excitation at their fundamental and harmonic modes^19–22^. Hence, the generation of low- and high-frequency ultrasound from a single element by patterning its driving electrodes into several segments and activating different modes with an electrical frequency-switchable control unit is possible. To manage the required frequency bands and optimize the corresponding vibrational modes, the design principle of patterned electrodes and their driving method have been explored. For example, Wang et al. presented individual five-electrode configurations in a single rectangular membrane PMUT^22^. By activating different electrode sets, the synthesized in-phase motion part enabled the PMUT to vibrate in the 1st, 3rd, and 5th modes, producing ultrasound at corresponding frequencies of 2.01, 3.19, and 5.84 MHz. Dual electrodes have also been used in an annular form for circular membrane devices. For example, Wu et al. introduced two ring-type electrodes in a circular PMUT that was designed to operate in the (0,1) and (0,2) modes at 3.75 and 18 MHz, respectively^19^. Here, by optimizing the design parameters (including the electrode’s width and position), vibrational crosstalk between the two resonant modes of the diaphragm could be eliminated. These design strategies successfully extended the available frequency band of a single device, showing the potential for advanced biomedical imaging. Although this is a promising method for achieving multiband frequencies from harmonic modes, the number of interconnections for patterned electrodes needs to be considered. As the number of elements increases to achieve better performance, individually addressing the elements derived from massive interconnections becomes challenging.

This paper reports a novel and simple method for generating dual-frequency ultrasound from a uniformly designed PMUT array. The presented method is established on a polarization state that is dependent on the vibrational motion of a ferroelectric PMUT, which only requires a single membrane and a driving electrode to cover the separated dual frequency bands. Moreover, by tuning the polarization state of the ferroelectric film using DC bias, the two types of driving modes can be switched. These modes cause the ferroelectric PMUT to emit low-frequency (5 MHz) and high-frequency (10 MHz) ultrasounds from a single excitation frequency of 5 MHz. The first section of the paper presents the concepts of dual frequency generation in a ferroelectric PMUT and demonstrates the interrelationship between the vibrational motion of the PMUT and the polarization state of the ferroelectric film. From this PMUT behavior, we propose a method of generating dual-frequency ultrasound from a single device by adjusting the DC bias. For proof of concept, a ferroelectric Pb(Mg_1/3_Nb_2/3_)O_3_-PbZrO_3_-PbTiO_3_ (PMN-PZT) thin-film-based PMUT array was manufactured using microfabrication techniques. Subsequently, sufficient driving conditions for each mode (low and high frequency) were investigated by measuring the acoustic pressure in a fluid under varying DC biases and AC amplitudes. In terms of potential use as a future imaging device, safe conditions for self-heating PMUT and potential skin burns were investigated. Furthermore, the zoom-in and zoom-out capabilities of the frequency-tunable PMUT are presented through B-mode imaging of the wire phantoms.

## Concepts of dual frequency generation in ferroelectric PMUTs

The proposed approach for generating dual-frequency ultrasound was established from the unique vibration mode of a PMUT, which strongly depended on the polarization state of its ferroelectric film. Different the proposed technique, conventional driving methods have transducers that uses either a nonferroelectric film (such as aluminum nitride (AlN)^23–25^) or a strongly polarized ferroelectric film; this transducer operates below the coercive voltage (the voltage required to induce domain reversal). Here, driving with a large bipolar signal above the coercive voltage results in the membrane vibrating at a frequency that is desynchronized from the input voltage.

**Fig. 1:**
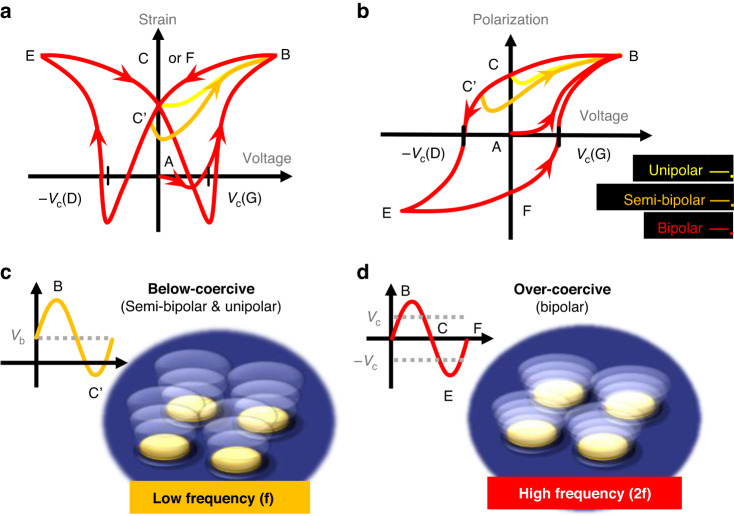
Conceptual explanation of dual frequency generation in ferroelectric PMUT. **a** Illustration of the general strain-voltage curve for ferroelectric film. **b** Illustration of the general polarization–voltage curve for ferroelectric film. **c** High frequency operation of the ferroelectric PMUT. **d** Low frequency operation of the PMUT

Figure 1a and b present the general polarization and strain curves for ferroelectric materials as a function of input voltage^26–29^. In ferroelectrics, such as PZT, PMN-PT, and PMN-PZT, a single driving cycle at a voltage level above the coercive voltage results in two electromechanical displacement cycles of the membrane^30^. At segment A → B (red lines in Fig. 1a and b), the film is not yet polar and has started to be polarized in the thickness direction; here, the electromechanical-induced stress is contractive in the transverse direction, forcing the membrane to flex upward^31^. In contrast, as the polarization decreases with a negative sign of the derivative of the voltage (B → D), the membrane attempts to switch the direction of the motion. At segment C → D, the voltage polarity is opposed to the remaining polarization in the film, and the tensile stress strongly forces the membrane to move down. This series of movements occurs for both signs of the bipolar cycle (A → D and D → G). Consequently, the periodical reversal of polarization during every excitation cycle causes two cycles of flextensional vibration in the membrane, resulting in the output transmit frequency being twice that of the frequency of the input voltage (f_out_ = 2 f_in_). Conversely, in the cases of unipolar (yellow line) or semibipolar (orange line) driving, unidirectional stress occurs in cycles due to the consistent orientation of the polarization. This results in a vibrational frequency (f_out_ = f_in_) of the PMUT that is synchronized with the driving voltage.

Dual-frequency ultrasound transmission using DC bias is proposed based on the nonlinear vibrational behavior of PMUTs, which is derived from polarization hysteresis. For the low-frequency (f_out_ = f_in_) emissions, a large DC bias is also applied to ensure that the ferroelectric film is strongly poled and that domain reversal can be restrained during the driving cycles (Fig. 1c). Conversely, to generate high-frequency (f_out_ = 2 f_in_) ultrasound, the PMUT is driven by large bipolar swings at levels above the coercive voltage without any bias (or with a weak DC bias), enabling easy domain switching (Fig. 1d). In both modes of operation, only a single driving frequency (f_in_) is needed.

This approach uses a different mechanism compared to the conventional method of applying DC bias for frequency tuning of PMUTs. The existing method mainly relies on utilizing the DC voltage generated prestress of the membrane, which in turn alters the flexural rigidity of the membrane and consequently changes the natural frequency and mode shape of the PMUT^32^. In contrast, our proposed method harnesses the unique characteristics of ferroelectric materials and polarization switching in the film, resulting in vibrational frequency doubling of the membrane, which has not yet been reported.

## Materials and methods

### Design and fabrication of the PMUT array

**Fig. 2:**
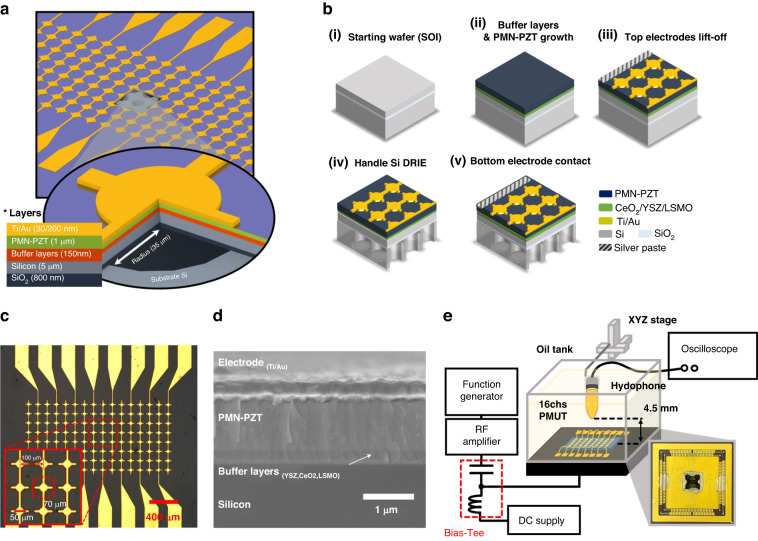
1D PMN-PZT PMUT device for dual frequency generation. **a** Schematic of the 1-dimensional array of the PMN-PZT PMUT. **b** Fabrication process of the 1-dimensional array of the PMN-PZT PMUT. **c** Optical image of the PMN-PZT PMUT. **d** Cross-sectional SEM image of the PMN-PZT film on silicon. **e** Illustration of the acoustic experimental set-up

The PMUT is designed as a 1-dimensional array for acquiring B-mode ultrasound images. Utilizing multiple elements enables 2-dimensional ultrasound imaging without the need for mechanical scanning. As illustrated in Fig. 2a, it consists of 16 independently drivable elements. Each element contains circular PMUT cells with a diameter of 70 μm. The pitch of the elements is 100 μm. A cross-sectional schematic of the PMUT demonstrates its composition. The PMUT is composed of a PMN-PZT piezoelectric layer. PMN-PZT is a ferroelectric material that exhibits spontaneous polarization and can be switched by an external electric field. For the common ground electrode necessary for the d_31_ operation of the PMUT, a La_0.67_Sr_0.33_MnO_3_ (LSMO) layer is utilized. LSMO is a conductive perovskite oxide, serves as a ground electrode and aids in the growth of PMN-PZT due to its similar crystal structure^33^. To simplify the fabrication process, the PMUT is constructed using an SOI wafer, with a silicon device layer serving as the PMUT’s passive layer.

An overall fabrication process flow of the PMUT is presented in Fig. 2b. The fabrication process was started by sequentially growing three buffer layers: yttria-stabilized zirconia (YSZ), cerium oxide (CeO_2_), and LSMO. These were grown on a 5 μm-thick device layer of silicon on an insulator (SOI) wafer using pulsed laser deposition (PLD). This oxide heterostructure bridged perovskite ferroelectric film and Si with structural similarities (symmetry and lattice parameters), enabling the growth of a film with high crystalline quality^34,35^. The laser energy density for deposition was set to 1.5 J/cm^2^, and each layer was grown to the following thicknesses at 750 °C: 50 nm (YSZ), 50 nm (CeO_2_), and 75 nm (LSMO).

After deposition of the buffer layers, the (001)-oriented ferroelectric PMN-PZT film was grown by sputtering with a 3:1 ratio of Ar and O_2_ gases at 600 °C (Fig. 2b(ii)). On the grown 1 μm thick PMN-PZT film, which is shown in the cross-sectional SEM image of the PMUT (Fig. 2c), a bilayer of Ti/Au (30 nm/200 nm) was deposited using sputtering and then patterned via lift-off (Fig. 2b(iii)). Subsequently, 70 μm diameter circles were patterned on the handle silicon layer of the SOI wafer using lithography and then etched using deep-reactive ion etching to define the PMUT membrane (Fig. 2b(iv)). Figure 2c displays an optical top view of the 1-dimensional (1D) PMUT array with the circular cells. The PMUT array consisted of 16 elements that could be driven individually and 8 cells within each element that were electrically linked together, resulting in simultaneous excitation of all cells in each element. Finally, for electrical access to the grounded LSMO layer, the PMN-PZT layer on the edge of the PMUT chip was physically peeled off, and conductive silver paste was applied (Fig. 2b(v)).

### Electrical characterization of PMN-PZT film

The hysteresis (polarization vs. voltage) loops of the (001)-oriented ferroelectric PMN-PZT thin film on PMUT were characterized at room temperature (25 °C) using a ferroelectric test system (Precision Premier ii, Radiant Technologies). Here, a cyclical bipolar voltage ranging from 2 to 80 V_pp_ was applied to the PMN-PZT film at 1 mHz; this was the lowest sweeping frequency in the measurement system. The polarization properties of the ferroelectric film depended on the sweeping frequency. Therefore, to determine the DC voltage conditions for polarization control, we utilized the lowest frequency available. Each loop was obtained after five sampling cycles to average out the noise.

### Acoustic characterization of PMUT

The acoustic pressure output of the ferroelectric PMUT was measured in a liquid environment (soybean oil) with the following characteristics: sound speed c of 1466 m ∙ s^−1^ at room temperature^36^ and mass density of 917 kg∙m^−3^^37^. This vegetable oil has been widely used as a coupling medium in acoustic imaging studies because it has similar acoustic properties to human tissue and good electrical insulation characteristics^38^. In the experiments, the PMUT was wire bonded to a ceramic pin grid array and electrically connected to the driving system using a customized printed circuit board. The transducer was driven by sinusoidal pulses of up to 80 V_pp_, which were generated by a function generator (33500B, Keysight) and magnified by an RF amplifier (325LA, Electronics & Innovation) in sequence. To investigate the effect of the DC bias on the output pressure of the PMUT, a bias voltage ranging from −20 to +20 V was applied using a power supply (E36234A, Keysight) and a bias network (5575 A Bias Tee, Picosecond). As a function of the DC bias and the magnitude of the AC pulses, the output pressure generated from the PMUT was measured using a calibrated hydrophone (HGL-0200, ONDA) at a distance of 4.5 mm from the PMUT surface, as displayed in Fig. 2e. The one-way travel time of the ultrasound was ~3 μs. The recorded acoustic signals were then decomposed into their frequency components using a fast Fourier transform (FFT). In the diverse driving conditions of the results, the correlation between polarization dynamics in the ferroelectric film and the PMUT output pressure was thoroughly examined, and the optimal conditions for dual-frequency ultrasound generation were derived from the frequency response of the transmitted ultrasound.

### Thermal dissipation measurement

In the high-frequency ultrasound operation mode, overcoercive voltage driving can cause self-heating problems associated with electrical losses of the ferroelectrics^39,40^. Moreover, repetitive switching polarization during excitation can induce dielectric losses, which are derived from the domain wall moving^41^. In turn, this loss results in heat dissipation of the ferroelectric film that is deposited within the transducer itself, restricting its practical use as a biomedical imaging transducer due to the possibility of burning the skin. Accordingly, the thermal dissipation from the PMUT was investigated under a range of tone-burst operating conditions. With an established optimal condition of DC bias for high-frequency ultrasound, the PMUT was driven by 10 cycles of a large AC swing (80 V_pp_), which was sufficient to fully switch the polarization orientation. The temperature increases at duty cycles from 1 to 20% were observed by capturing thermographic images of the oil-immersed PMUT using an infrared camera (FLIR A325sc, FLIR systems). In each case, the corresponding pulse repetition frequency (PRF) was set to 5, 25, 50, and 100 kHz.

### Ultrasound imaging with PMUT

The ultrasound imaging performance of the PMUT was assessed in both modes of operation by obtaining B-mode images of wire phantoms using an ultrasound research platform (Vantage 64, Verasonics). To obtain two images, a DC voltage of 20 V (in low frequency mode) and −2.3 V (in high frequency mode) were applied to the PMUT, and an AC voltage of 70 Vpp with a single cycle was applied. Here, 5 copper wires with a diameter of 100 μm were equally spaced at 2 mm and distributed at depths of 6–14 mm from the PMUT surface. In the imaging setup, all elements in the 1-D array were used for transmission and reception of the ultrasound signals. The transmitted wave steered from −20° to +20° in 31 sequential transmission events by adding a gradual delay to the firing time in each element. During the receiving events, backscattered ultrasound echoes from the plane wave emitted at different steer angles were recorded, processed, and beamformed in parallel by implementing synthetic dynamic focus to reconstruct the image^42^. This coherent plane-wave compound (CPWC) technique provides a higher SNR of the image compared to synthetic aperture imaging (SAI) due to the large array gain during transmission. Furthermore, CPWC provides a high frame rate with the same quality as conventional multifocal B-mode imaging^43,44^.

## Results and discussion

### DC bias effects on ultrasound frequency

**Fig. 3:**
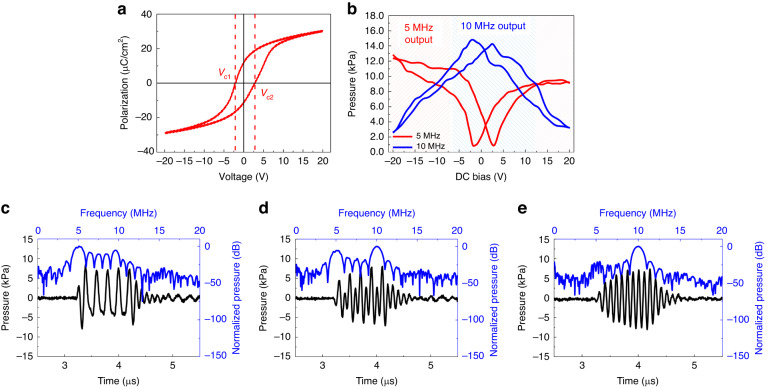
Acoustical characterization of the dual-frequency 1D PMN-PZT PMUT. **a** Polarization–voltage curve of grown PMN-PZT film. **b** Output pressures of 5 and 10 MHz frequencies under varying DC biases from −20 to +20 V. **c** Output pressure when driving with 20 Vdc. **d** Output pressure when driving with 10 Vdc. **e** Output pressure when driving with −2.3 Vdc

The grown PMN-PZT film had almost symmetrical polarization characteristics with respect to the zero voltage axis. Figure 3a displays the hysteresis curve of the No. 5 element in the PMUT array. Here, the measured coercive voltages where polarization reversal occurred were −2.3 and +2.5 V under negative and positive biases, respectively. The remanent polarization was ± 17 μC/cm^2^. To demonstrate the dependency of the ultrasound frequency on the polarization state of the ferroelectric film, the ultrasound output signals from the PMUT were investigated under three different bias points, which represented the polarization of PMN-PZT. Figure 3c–e shows the output ultrasound signals at 20 V (strongly poled in an upward direction), 10 V (weakly poled), and −2.3 V (not polarized with negative coercive bias). Under these biases, the driving pulse was set to 5 MHz (5 cycles) and an AC voltage magnitude of 30 V_pp_ for all output ultrasound signals. In the strongly poled state with high bias (Fig. 3c), the unipolar driving did not switch the polarization direction during the excitation cycles. The unidirectional induced piezoelectric stress resulted in synchronized vibrational motion of the membrane and an emitted ultrasound frequency identical to the driving signal (5 MHz).

As the polarization was weakened by reducing the bias, polarization reversal during the negative half-driving cycle started to switch the direction of the piezoelectric stress and caused the PMUT to vibrate faster. Consequently, the accelerated vibration of the PMUT resulted in second-harmonic ultrasound generation with a frequency that was double the driving signal (10 MHz), as displayed in Fig. 3d. When the bias point reached the coercive voltage (−2.3 V), the polarization was easily reversed by the bipolar excitation, resulting in maximized harmonic ultrasound components (Fig. 3e). This frequency shift was also exhibited in the upward sweeping bias, with the exception of the coercive voltage. If the ferroelectric film was strongly poled in the downward direction with a negative bias, the PMUT emitted a 5 MHz ultrasound signal. As the polarization weakened due to the upward sweeping bias, high-frequency (10 MHz) ultrasound became intensive and maximized at a positive coercive voltage of 2.5 V. These characteristics resulted in an inverse butterfly shaped loop for the relationship between the DC bias and 10 MHz ultrasound components (blue solid line in Fig. 3b). The low-frequency ultrasound (5 MHz) manifested in the opposite manner, showing a butterfly shaped loop (red solid line in Fig. 3b). This was minimized at coercive biases (−2.3 and +2.5 V) and started to increase with the high bias voltage in both directions. In conclusion, the PMUT’s driving modes for low- and high-frequency ultrasound could be easily switched by tuning the DC bias. For the low-frequency ultrasound, a high bias was needed and caused the ferroelectric to be strongly poled (red dashed area in Fig. 3b). Conversely, a low bias near the coercive voltages was necessary to obtain the high-frequency ultrasound (blue dashed area in Fig. 3b).

### AC swing for high-frequency ultrasound

**Fig. 4:**
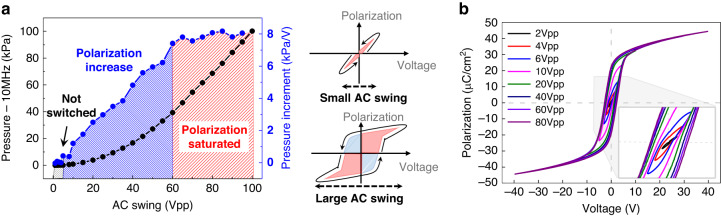
Acoustical characterization of the 1D PMN-PZT PMUT in high frequency operation. Conditions of the AC swing voltage for the high frequency ultrasound: (**a**) 10 MHz Output pressures generated from the PMUT in high frequency operation. **b** Ferroelectric polarization hysteresis for a PMN-PZT thin film with applied voltage levels of 2, 4, 6, 10, 20, 40, 60, and 80 V_pp_

In addition to coercive biasing, for high-frequency ultrasound generation, the PMUT needs to be driven with a large bipolar signal that can fully induce ferroelectric domain switching^31^. This concept is demonstrated in Fig. 4, which shows the transmitted pressure from the PMUT (Fig. 4a) and polarization hysteresis (Fig. 4b) under varying driving voltages. During the experiments, a range of AC voltages from 2 to 80 Vpp, with a DC voltage of −2.3 V corresponding to the coercive voltage, was applied. This simultaneous application was essential for ensuring the proper operation of the high-frequency mode. As presented in the gray dashed area of Fig. 4a, the small AC swing below 6 V_pp_ was unable to force the PMUT to sufficiently vibrate, resulting in the emitted pressure being too low to detect. Under low excitations, polarization in the PMN-PZT film did not exhibit hysteresis, resulting in no domain switching^45^. However, above this threshold value of 6 V_pp_, the transmitted pressure rapidly increased due to significant hysteresis, resulting in sufficient domain switching (blue dashed area of Fig. 4a). At voltage levels above 60 V_pp_, the polarization of the PMN-PZT started to saturate, resulting in constant piezoelectricity^32^. Under these large swings, the PMUT exhibited a linear relationship between the driving voltage and output pressure (red dashed area of Fig. 4a).

### Heat dissipation during the overcoercive driving mode

**Fig. 5:**
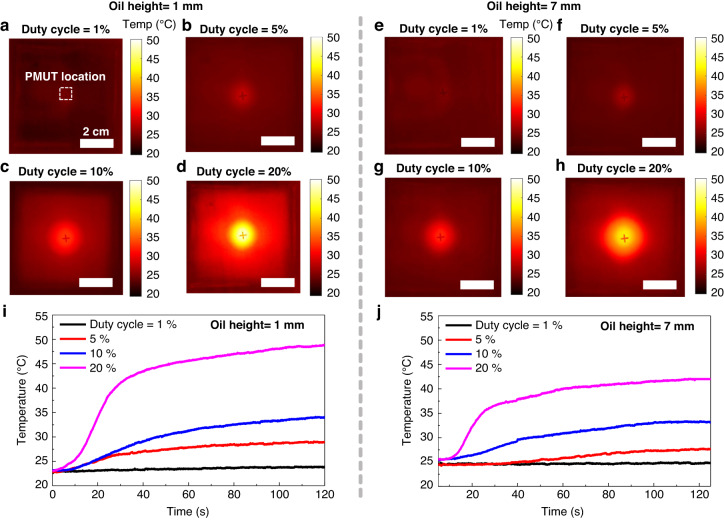
Thermal infrared images of oil-immersed PMUT when it was driven by an overcoercive voltage swing. Each image was captured at 120 s after driving with various duty cycles and oil heights. The driving voltage was identical: 5 MHz, 10 cycles, and 80V_pp_ with −2.3 Vdc coercive voltage. The oil height was 1 mm for the following duty cycles: (**a**) 1%, (**b**) 5%, (**c**) 10%, and (**d**) 20%. The oil height was 7 mm in the following duty cycle cases: (**e**) 1%, (**f**) 5%, (**g**) 10%, and (**h**) 20%. Temperature‒time profiles at center point of the oil-immersed PMUT in the case of oil heights (**i**) 1 mm and (**j**) 7 mm

The aim of this study was to evaluate the self-heating characteristics of PMN-PZT PMUT under an overcoercive driving mode to achieve high-frequency ultrasound generation. The periodical domain wall motion resulted in dielectric losses on each excitation cycle and heat dissipation from the driven PMUT. The duty cycle, which is an excitation parameter, was mainly investigated to obtain the safe driving conditions to avoid self-heating and predictable thermal injuries when used as imaging transducers^46^. In the experiments, the PMUT was driven at a large AC voltage of 80 V_pp_ and −2.3 V coercive biasing, which was the established condition from the preceding experiments (Fig. 5). As demonstrated in the thermal infrared images of an oil-immersed PMUT with an oil height of 1 mm (captured at 120 s after firing the PMUT), the temperature of the PMUT drastically increased with increasing duty cycles due to insufficient cooling time. For the 20% duty cycle (the highest value in the experiments), the temperature increased to 49 °C and became saturated. This temperature indicated that a duty cycle above 20% would either restrict medical usage (due to potential skin burns) or require safe time limits (~19 s below 35 °C)^47^.

The temperature increase could be alleviated by increasing the oil (acoustic media) height. When the oil height was increased to 7 mm, the temperature saturated at 42 °C, which was 17% lower than when the height was 1 mm. This result was due to the heat dispersion of the surrounding oil. In addition, the safe usage time was extended to ~25 s below 35 °C. In addition, the saturated temperatures did not exceed 35 °C in either case at duty cycles of 1%, 5%, and 10%, indicating that it could be used without any safety concerns. Typically, pulse-echo imaging schemes prefer low-duty cycles, except for special cases, such as fast Doppler imaging, that need to detect high-velocity blood flows without aliasing^48^ because short pulse duration and low pulse repetition frequency (PRF) result in enhanced axial resolution and large imaging depth^49^.

### Ultrasound imaging

**Fig. 6:**
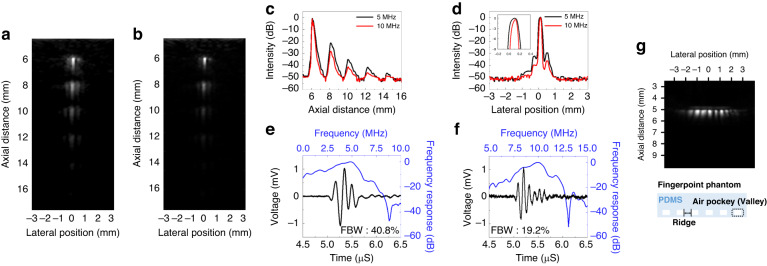
Ultrasound B-mode images of the wire phantoms (0.1 mm diameter) acquired with Vantage 64 system. The five wire phantoms were located at depths of 6, 8, 10, 12 and 14 mm in the oil: (**a**) 30 dB compressed image obtained from 5 MHz ultrasound sonication when 20 Vdc was applied to the PMUT. **b** 30 dB compressed image obtained from 10 MHz ultrasound sonication when −2.3 Vdc (Coercive voltage) was applied to the PMUT. **c** Axial beam profiles obtained from the wire targets. **d** Lateral beam profiles obtained from a wire located at 6 mm depth. **e** Ultrasound transmit signal from the low frequency mode PMUT produced a single cycle pulse excitation. **f** Ultrasound transmit signal from the high frequency mode PMUT. **g** 30 dB compressed B-mode image of the PDMS fingerprint phantom

With ultrasound imaging, the penetration depth and spatial resolution strongly depend on the ultrasound wave’s frequency. Compared to low-frequency pulses, high-frequency pulses yield a narrow beamwidth and a resultant improved spatial resolution at the expense of a shallow depth of penetration due to high attenuation^50^. Figure 6a displays a 30 dB compressed grayscale ultrasound image of the wire phantoms obtained by 5 MHz ultrasound, and Fig. 6b displays an image from a 10 MHz ultrasound signal. As demonstrated in the images, the high frequency (10 MHz) ultrasound enhanced lateral resolution and reduced imaging depth compared to the image obtained using the 5 MHz ultrasound. The measured full-width half maximum on the lateral axis of the 6 mm located wire was estimated as 155 μm in the 10 MHz ultrasound image, which was 21% smaller than that of the 5 MHz image (196 μm), as shown in Fig. 6c. However, the wire at 14 mm depth in the 10 MHz ultrasound image could not be clearly observed due to the rapidly decreasing signal-to-noise ratio of 5.1 dB. The noise floor was −53.7 dB, and the intensity reflected from the wire was −45.8 dB. In contrast, the SNR of the 14 mm wire in the 5 MHz image was 7.9 dB and remained distinguishable.

There was no significant difference in axial resolution (the capability of resolving two reflectors along the beam’s path) between images. This occurred because the pulse duration of high-frequency ultrasound generated from the overcoercive mode of the PMUT was comparable to that of low-frequency ultrasound. When the PMUT was subjected to overcoercive driving, the membrane vibrated in twice as many cycles compared to when operating in normal driving mode for low frequencies. The axial resolution in ultrasound imaging is determined by the total pulse duration (μsec), which is a product of the period (nsec) and number of cycles (#). From this observation, the comparison of pulse duration between the two modes of the PMUT further supports these findings. Figure 6e and f depict the results of pulse width and bandwidth for the low-frequency and high-frequency modes, respectively. When a 5 MHz pulse with a single cycle of 70 Vpp was applied, the low-frequency mode exhibited a 40.8% −3 dB fractional bandwidth (FBW) centered at 5 MHz. Conversely, the high-frequency mode, with twice the number of vibrational cycles, had a narrower FBW of 19.2%.

To mitigate these technical limitations, we aimed to leverage the PMUT for fingerprint imaging in a specific application. In fingerprint and similar surface imaging, the lateral resolution is often more crucial than the axial resolution. Since there was no significant difference in axial resolution between the images due to comparable pulse durations, we focused on the potential to enhance lateral resolution.

In Fig. 6g, the B-mode image fingerprint phantom is shown. The fingerprint phantom was created using polydimethylsiloxane (PDMS). In line with the fundamental principle of imaging based on the impedance difference between ridges and valleys of a fingerprint, we mimicked the finger valleys by using air pockets (340 Rayls) with a significant impedance contrast to the acoustic medium, vegetable oil (1.5 MRayls), and the ridges were mimicked using PDMS (1.0 MRayls) with a smaller impedance difference. The pattern width and spacing were set to 200 μm, representing the average spacing between ridges and valleys in a human fingerprint. The acquired B-mode images clearly showed small reflection signals from the ridges and large ultrasound reflection signals from the valleys. The difference between these two reflection signals was ~23.3 ± 2.6 dB, demonstrating a distinct separation.

## Conclusions

Our study presented a novel method for dual-frequency ultrasound generation in ferroelectric PMUTs using a single excitation frequency (5 MHz). The ferroelectric PMUT exhibited a shift in output ultrasound frequency under varying DC biases and resultant polarization states of the ferroelectric film. At a high DC bias (positive or negative polarity), the ferroelectric film was strongly poled, and direction switching did not occur during excitation. This resulted in the synchronization of the output ultrasound frequency and driving signal (5 MHz). Moreover, at 10 MHz, a second harmonic ultrasound of the input frequency was produced by a large AC swing (>6 V_pp_) at coercive voltage biases of −2.3 V (negative polarity) or +2.5 V (positive polarity), which induced the movement of the periodical domain wall in the ferroelectrics. Accordingly, driving mode switching by tuning the DC bias provided the option to select the frequency for better imaging resolution and depth, resulting in a more comprehensive understanding of the anatomic structure of targets. For future biomedical imaging applications, driving conditions that can avoid predictable thermal injuries to the subject will be investigated. Sufficient cooling time below 10% duty cycles warrants safe usage without any self-heating problems of the transducer. Moreover, this safety could be improved by packaging the PMUT with higher thermally conductive media, such as polyimide and silicon rubber compounds (RTV 615 and 655); these compounds are well-established materials for producing the acoustic lenses of imaging transducers. This media can help to mitigate any increasing heat on the transducer since their thermal conductivity of 0.2–0.5 W/mK is higher than that of the soybean oil (0.153 W/mK) used in this study.

## Data Availability

The datasets generated during and/or analyzed during the current study are available in the [figshare] repository: [https://figshare.com/s/bd724150fbe0cf5a5adb].

## References

[1] Sun XL, Yan JP, Li YF, Liu H (2018). Multi-frequency ultrasound transducers for medical applications: a survey. Int. J. Intell. Robot. Appl..

[2] Liu HL, Hsieh CM (2009). Single-transducer dual-frequency ultrasound generation to enhance acoustic cavitation. Ultrason. Sonochem..

[3] Qiu W (2015). A novel dual-frequency imaging method for intravascular ultrasound applications. Ultrasonics.

[4] Ye, L., Zhu, X., & Liu, Y. Numerical study on dual-frequency ultrasonic enhancing cavitation effect based on bubble dynamic evolution. *Ultrason. Sonochem*. **59**, (2019). 10.1016/j.ultsonch.2019.104744. 10.1016/j.ultsonch.2019.104744 31473426

[5] Barati AH, Mokhtari-Dizaji M, Mozdarani H, Bathaie Z, Hassan ZM (2007). Effect of exposure parameters on cavitation induced by low-level dual-frequency ultrasound. Ultrason. Sonochem..

[6] Ye, L., Zhu, X., He, Y., & Song, T. Effect of frequency ratio and phase difference on the dynamic behavior of a cavitation bubble induced by dual-frequency ultrasound. *Chem. Eng. Process*. **165**, (2021). 10.1016/j.cep.2021.108448.

[7] Ma, J., et al., Dual frequency transducers for intravascular ultrasound super-harmonic imaging and acoustic angiography. *IEEE International Ultrasonics Symposium*, IUS 675–678 (IEEE Computer Society, 2014). 10.1109/ULTSYM.2014.0166.

[8] Ma, J., Wang, Z., Li, S., Jiang, X. Anti-matching design for wave isolation in dual frequency transducer for intravascular super-harmonic imaging. *International Mechanical Engineering Congress and Exposition, Proceedings (IMECE)* (American Society of Mechanical Engineers (ASME), 2014). 10.1115/IMECE2014-38844.

[9] Newsome IG (2021). Implementation of a Novel 288-Element Dual-Frequency Array for Acoustic Angiography: In Vitro and in Vivo Characterization. IEEE Trans. Ultrason. Ferroelectr. Freq. Control.

[10] Cai, Y., Luo, X., Xu, L., Chen, Z., & Ma, J. Broadband Stack-layer 3 MHz - 11 MHz Dual-frequency Ultrasound Transducers for Photoacoustic Imaging. *IEEE International Ultrasonics Symposium, IUS*, IEEE Computer Society (2022). 10.1109/IUS54386.2022.9958818.

[11] Fei, C., et al., Design of matching layers for high-frequency ultrasonic transducers. *Appl. Phys. Lett*. **107**, (2015). 10.1063/1.4931703. 10.1063/1.4931703 PMC4583513 26445518

[12] van Neer PLMJ (2010). Super-Harmonic Imaging: Development of an Interleaved Phased-Array Transducer. IEEE Trans. Ultrason. Ferroelectr. Freq. Control.

[13] Maadi M, Ceroici C, Zemp RJ (2021). Dual-Frequency CMUT Arrays for Multiband Ultrasound Imaging Applications. IEEE Trans. Ultrason. Ferroelectr. Freq. Control..

[14] Liu, L., et al., A dual-frequency piezoelectric micromachined ultrasound transducer array with low inter-element coupling effects. *J. Micromech. Microeng*. **31**, (2021). 10.1088/1361-6439/abde8f.

[15] Zheng Q (2022). Thin ceramic PZT dual- and multi-frequency pMUT arrays for photoacoustic imaging. Microsyst. Nanoeng..

[16] Pekař M, Dittmer WU, Mihajlović N, van Soest G, de Jong N (2017). Frequency Tuning of Collapse-Mode Capacitive Micromachined Ultrasonic Transducer. Ultrasonics.

[17] Wang, J., et al., A review on analytical modeling for collapse mode capacitive micromachined ultrasonic transducer of the collapse voltage and the static membrane deflections. *Micromachines* (Basel) **12**, (2021). 10.3390/mi12060714. 10.3390/mi12060714 PMC8235715 34207176

[18] Pekař M (2017). Preclinical Testing of Frequency-Tunable Capacitive Micromachined Ultrasonic Transducer Probe Prototypes. Ultrasound Med. Biol..

[19] Wu, L., Moridi, M., Wang, G., & Zhou, Q. Microfabrication and characterization of dual-frequency piezoelectric micromachined ultrasonic transducers. *IEEE International Symposium on Applications of Ferroelectric*, ISAF (2021). 10.1109/ISAF51943.2021.9477337.

[20] Cai J (2022). Beyond fundamental resonance mode: high-order multi-band ALN PMUT for in vivo photoacoustic imaging. Microsyst. Nanoeng..

[21] Sun, C. et al. Investigation of Broadband Characteristics of Multi-Frequency Piezoelectric Micromachined Ultrasonic Transducer (MF-pMUT. IEEE Sens. J. **19**, 860–867 (2019).

[22] Wang, T. & Lee, C. Electrically switchable multi-frequency piezoelectric micromachined ultrasonic transducer (pMUT). *IEEE International Conference on Micro Electro Mechanical Systems (MEMS)* 1106–1109 (Institute of Electrical and Electronics Engineers Inc., 2016). 10.1109/MEMSYS.2016.7421828.

[23] Jiang X (2017). Monolithic ultrasound fingerprint sensor. Microsyst. Nanoeng..

[24] Wang Q, Lu Y, Mishin S, Oshmyansky Y, Horsley DA (2017). Design, Fabrication, and Characterization of Scandium Aluminum Nitride-Based Piezoelectric Micromachined Ultrasonic Transducers. J. Microelectromec. Syst..

[25] Han C (2012). High potential columnar nanocrystalline AlN films deposited by RF reactive magnetron sputtering. Nanomicro. Lett..

[26] Boser O (1987). Statistical theory of hysteresis in ferroelectric materials. J. Appl. Phys..

[27] Luo J, Zhang S (2014). Advances in the growth and characterization of relaxor-PT-based ferroelectric single crystals. Crystals (Basel).

[28] Li, Y. W., Zhou, X. L., Miao, H. C., Cai, H. R., & Li, F. X. Mechanism of crystal-symmetry dependent deformation in ferroelectric ceramics: Experiments versus model. *J. Appl. Phys*. **113**, (2013). 10.1063/1.4809979.

[29] Wang, N., et al., Structure, Performance, and Application of BiFeO3 Nanomaterials. *Nanomicro. Lett*. **12**, (2020). 10.1007/s40820-020-00420-6. 10.1007/s40820-020-00420-6 PMC7770668 34138095

[30] Dausch DE, Castellucci JB, Chou DR, von Ramm OT (2008). Theory and operation of 2-D array piezoelectric micromachined ultrasound transducers. IEEE Trans. Ultrason. Ferroelectr. Freq. Control..

[31] Nguyen MD, Vu HN, Rijnders G (2021). Nonlinearity in inverse and transverse piezoelectric properties of Pb(Zr0.52Ti0.48)O3 film actuators under AC and DC applied voltages. Curr. Appl. Phys..

[32] Kusano Y (2018). Effects of DC Bias Tuning on Air-Coupled PZT Piezoelectric Micromachined Ultrasonic Transducers. J. Microelectromech. Sys..

[33] Choi HJ (2022). Thermal stress-assisted annealing to improve the crystalline quality of an epitaxial YSZ buffer layer on Si. J. Mater. Chem. C Mater..

[34] Hanzawa, H., Yoshida, S., Wasa, K., & Tanaka, S. Highly c-axis oriented monocrystalline Pb(Zr, Ti)O3 based thin film on Si wafer by sputter deposition with fast cooling process. *IEEE International Ultrasonics Symposium*, IUS, IEEE Computer Society (2014), 907–910 10.1109/ULTSYM.2014.0222. 10.1109/TUFFC.2014.3069 25167155

[35] Aytug T (2001). La0.7Sr0.3MnO3: A single, conductive-oxide buffer layer for the development of YBa2Cu3O7-δ coated conductors. Appl. Phys. Lett..

[36] Oliveira, P. A., Silva, R. M. B., Morais, G. C., Alvarenga, A. V., & Costa-Félix, R. P. B. Speed of sound as a function of temperature for ultrasonic propagation in soybean oil. *J. Phys. Conf. Ser*. Institute of Physics Publishing (2016). 10.1088/1742-6596/733/1/012040.

[37] Noureddini H, Teoh BC, Clements D (1992). Densities of Vegetable Oils and Fatty Acids. J. Am. Oil Chem. Soc..

[38] Filippou, A., Louca, I., & Damianou, C. Characterization of a fat tissue mimicking material for high intensity focused ultrasound applications. *J. Ultrasound* (2022). 10.1007/s40477-022-00746-4. 10.1007/s40477-022-00746-4 PMC10247632 36414928

[39] Liu G, Zhang S, Jiang W, Cao W (2015). Losses in ferroelectric materials. Mater. Sci. Eng. R: Rep..

[40] Barati, M., et al., Investigation of self-heating and dissipative effects in ferroelectric ceramics subjected to compressive mechanical cyclic loading. *Acta. Mater*. **221**, (2021). 10.1016/j.actamat.2021.117386.

[41] Malyshkina O, Eliseev A, Grechishkin R (2017). Heat losses in ferroelectric ceramics due to switching processes. Proc. Estonian Acad. Sci..

[42] Montaldo G, Tanter M, Bercoff J, Benech N, Fink M (2009). Coherent plane-wave compounding for very high frame rate ultrasonography and transient elastography. IEEE Trans. Ultrason. Ferroelectr. Freq. Control.

[43] Tanter M, Fink M (2014). Ultrafast imaging in biomedical ultrasound. IEEE Trans. Ultrason. Ferroelectr. Freq. Control..

[44] Jensen J, Stuart MB, Jensen JA (2016). Optimized Plane Wave Imaging for Fast and High-Quality Ultrasound Imaging. IEEE Trans. Ultrason. Ferroelectr. Freq. Control.

[45] Zhou D (2015). Electric field and temperature scaling of polarization reversal in silicon doped hafnium oxide ferroelectric thin films. Acta. Mater..

[46] Shi, H., Chen, Z., Chen, X., Liu, S., & Cao, W. Self-heating phenomenon of piezoelectric elements excited by a tone-burst electric field. *Ultrasonics* **117**, (2021). 10.1016/j.ultras.2021.106562. 10.1016/j.ultras.2021.106562 34469832

[47] Yarmolenko PS (2011). Thresholds for thermal damage to normal tissues: An update. Int. J. Hyperthermia.

[48] Oglat AA (2018). A review of medical doppler ultrasonography of blood flow in general and especially in common carotid artery. J. Med. Ultrasound.

[49] Silverman RH (2009). ultrasound imaging of the eye - A review. Clin. Exp. Ophthalmol..

[50] Treeby BE, Zhang EZ, Thomas AS, Cox BT (2011). Measurement of the Ultrasound Attenuation and Dispersion in Whole Human Blood and its Components From 0-70 MHz. Ultrasound Med. Biol..

